# The Effectiveness of Selective Serotonin Reuptake Inhibitors for Treatment of Obsessive-Compulsive Disorder in Adolescents and Children: A Systematic Review and Meta-Analysis

**DOI:** 10.3389/fpsyt.2019.00523

**Published:** 2019-08-06

**Authors:** Vijaya Padma Kotapati, Ali M. Khan, Sara Dar, Gulshan Begum, Ramya Bachu, Mahwish Adnan, Aarij Zubair, Rizwan A. Ahmed

**Affiliations:** ^1^Department of Mental Health, Manhattan Psychiatric Center, Manhattan, NY, United States; ^2^Department of Mental Health, University of Texas Rio Grande Valley Edinburg, Edinburg, TX, United States; ^3^Department of Mental Health, Brigham and Women’s Hospital, Harvard Medical School, Boston, MA, United States; ^4^Department of Mental Health, Jamaica Hospital Medical Center, Richmond Hill, NY, United States; ^5^Department of Mental Health, Zucker Hillside Hospital, Glen Oaks, NY, United States; ^6^Department of Mental Health, Mc Master University, Ontario, CN, Canada; ^7^Department of Mental Health, St. Johns University, Queens, NY, United States; ^8^Department of Mental Health, Liaquat University of Medical & Health Sciences, Sindh, Pakistan

**Keywords:** selective serotonin reuptake inhibitors, obsessive-compulsive disorder, adolescents, children, cognitive behavioral therapy

## Abstract

**Background:** Obsessive-compulsive disorder (OCD) is a common behavioral disorder among adolescents and children. The selective serotonin reuptake inhibitors (SSRIs) are the first pharmacological choice for this condition due to mild adverse effect profile.

**Objective:** This systematic review was performed to evaluate the efficacy of SSRI for OCD in adolescents and children.

**Methods:** Search terms were entered into PubMed, PsycINFO, Scopus, CINAHL, and Google Scholar. The included studies were randomized, placebo-controlled trials of SSRIs conducted in populations of children and adolescents younger than 18 years. Change from baseline Children’s Yale-Brown Obsessive-Compulsive Scale (CY-BOCS), end-treatment CY-BOCS with respective SD, and response and remission rates were collected for continuous and dichotomous outcome assessment, respectively. Cochrane Rev Man software was used for meta-analyses, providing Forest plots where applicable.

**Results:** SSRIs were superior to placebo with a small effect size. There was no additional benefit of combination treatment over cognitive behavioral therapy (CBT) alone, but CBT added substantial benefit to SSRI monotherapy. Fluoxetine and sertraline appear to be superior to fluvoxamine.

**Conclusion:** The results of current systematic review and meta-analysis support the existing National Institute for Health and Care Excellence (NICE) guidelines for choosing CBT as first line of treatment and substituting it with SSRI, depending on patient preference. Adding CBT to current SSRI treatment is effective for non-responders and partial responders, but adding SSRI to ongoing CBT does not prove beneficial. The SSRIs have different effectiveness, and their relative efficacy remains to be investigated.

## Introduction

Obsessive-compulsive disorder (OCD) is a chronic debilitating condition that is associated with recurrent and persistent thoughts and the compulsions to suppress them with certain excessive and repetitive behaviors. For about half of the diagnosed cases, the onset of OCD takes place in childhood or adolescence (1). Compared with adults, children are more likely to demonstrate the evolution of clinical manifestations and the symptoms wax and wane as they grow (2, 3).

Currently, the diagnosis is based on internationally accepted classification systems, namely *Diagnostic and Statistical Manual of Mental Disorders* in its fifth edition (DSM 5) in the United States and, less frequently, ICD 10 criteria elsewhere. In addition to diagnosing, the severity of the condition is assessed and documented using validated scales, such as Children’s Yale-Brown Obsessive-Compulsive Scale (CY-BOCS) for children and adolescents, which is a modification of the original Yale-Brown Obsessive-Compulsive Scale (Y-BOCS) used for adults.

Medications and psychotherapy or a combination of both are commonly used to treat patients with OCD. The only currently available treatment options for OCD are either medication or psychotherapy or the combination thereof (4, 5), the latter being chosen for more severe, refractory to treatment and comorbid cases.

Treatment with clomipramine, a tricyclic antidepressant (TCA) that inhibits reuptake of serotonin, was the first option demonstrated to be effective at reducing OCD symptoms (6), and this was later confirmed in a 2003 meta-analysis (7) for the pediatric population. Although clomipramine is an effective option, it cannot be used as a first-line agent for treating OCD in children and adolescents due to its overburdened adverse effect profile (8, 9). Although it most commonly causes a combination of minor cholinergic symptoms, such as sedation, bothersome xerostomia, constipation, and urinary retention, and even more, severe events like seizures and cardiovascular effects, such as orthostatic hypotension, tachy- and bradyarrhythmias, ventricular fibrillation, and prolonged QT are well documented. In some of the devastating cases, sudden cardiac death in youth is attributed to such effects. Thus, a better tolerated and safer alternative has been sought to replace clomipramine as the more appropriate pharmaceutical candidate. The selective serotonin reuptake inhibitors (SSRIs) seem to efficiently fill in that role.

Approved by the FDA at the end of 1978, fluoxetine (Prozac) was the first agent marketed as an SSRI, and its use has once and for all established the role of serotonin (5-HT) in the pathogenesis of psychiatric disorders. SSRIs were in many aspects’ superior to the pre-existing classes of anti-depressants, i.e., TCAs and monoamine oxidase inhibitors (MAOIs). Being selective in nature of their action, SSRIs had a much more favorable side-effect profile (especially regarding that of arrhythmias and QT prolongation which were absent with the latter agents), easier dose titration, remarkable margin of safety when considering overdoses, and thus overall were better tolerated and adhered to by the populations with psychiatric and mood disorders (10).

A meta-analysis suggested clomipramine to be more effective compared with SSRI for the treatment of OCD in children (11). That said, direct comparisons of clomipramine and SSRI have not shown any superiority for any of the two drugs for treating OCD in adults (12–15). There has not been a single study comparing clomipramine to SSRI in a head to head design for treating OCD in children, and the claims of the former being more efficacious than the latter cannot be given considerable weight until proven otherwise.

The commonly held opinion is that cognitive behavioral therapy (CBT) should be the first line of treatment for OCD in children and adolescents. This approach has also been endorsed by the existing clinical guidelines (16, 17) and has a considerable base of evidence. A systematic review of 13 randomized controlled trials (RCTs) assessing the treatment options for OCD (18) pointed out to the place and efficacy of CBT for the management of OCD in children and adolescents, but the comparisons were made to waitlist and placebo treatment, and not to other available active options.

Thus, the current role of SSRIs in managing OCD in adolescents and children remains at least under-investigated. We have undertaken this review to address some of the gaps concerning SSRI treatment, as described more thoroughly below.

To be familiar with the ground of research on OCD treatment, the reader is humbly referred to the previously conducted systematic reviews and meta-analyses concerning the above-mentioned issues (7, 11, 19–23).

## The Rationale for This Review

The current systematic review was undertaken for the following reasons. There have been a number of both remote and recent systematic reviews and meta-analyses on various treatment options for OCD in adolescents and children (see above), including CBT, SRI, and the combination thereof, but none has specifically addressed the role of SSRIs, and comparisons between different SSRIs are lacking as well. Thus, we have decided to review the existing literature regarding treatments that utilize only SSRIs as the medication of intervention for treatment of OCD in adolescents and children.

## Aims and Objectives

The purpose of the current systematic review and meta-analysis was to investigate the efficacy of SSRIs in forms of monotherapy or in combination treatment with CBT for the management of OCD in adolescents and children and compare the effectiveness of different SSRIs.

## Methods

### Search for Publications

The authors (AK and VK) searched the PubMed, PsycINFO, Scopus, CINAHL, and Google Scholar databases using the following keywords: (“treatment” OR “therapy” OR “SSRI” OR “Selective Serotonin Reuptake Inhibitors” OR “sertraline” or “fluoxetine” OR “fluvoxamine “ OR “paroxetine” OR “citalopram” OR “escitalopram”) AND (“OCD” OR “obsessive compulsive disorder” OR “obsessional compulsive disorder”) AND (“children” OR “adolescents”), for citations from first available index date to January 1, 2018. The authors also manually searched the references from relevant systematic reviews and meta-analyses for additional citations that could have been missed through the initial search.

Two authors (AK and SD) then independently screened the titles and abstracts for the exclusion of irrelevant studies. The full papers were then obtained to verify for inclusion eligibility. At each step, the results were compared between the researchers and any discrepancies were handled with means of discussion. Any disagreements were resolved by involving the third independent author (RA).

### Eligibility for Inclusion

The studies were included if they successfully fulfilled any of the following criteria:

1. Participants were 18 years or younger
2. Participants had a primary diagnosis of OCD
3. SSRIs, such as escitalopram, fluoxetine, fluvoxamine, paroxetine, sertraline, vilazodone, and citalopram, were used
4. OCD symptom severity was reported *via* appropriate psychometric scale, i.e., CY-BOCS
5. RCTs
6. Studies published in English

### Data Synthesis

Change from baseline CY-BOCS, end-treatment CY-BOCS, and remission and response rates was compared for different interventions arms. Cochrane’s Review Manager software was used to perform effect size calculations *via* random effects model, and standard mean differences (SMDs) were presented for continuous and in odds ratios (ORs) for dichotomous outcomes, respectively. Forest plots and corresponding mean differences were presented in the figures and tables, where appropriate. A subgroup comparison was attempted for different SSRIs using the I^2^ statistic in generic inverse variance analysis.

## Results

### Search Results

**Figure 1:**
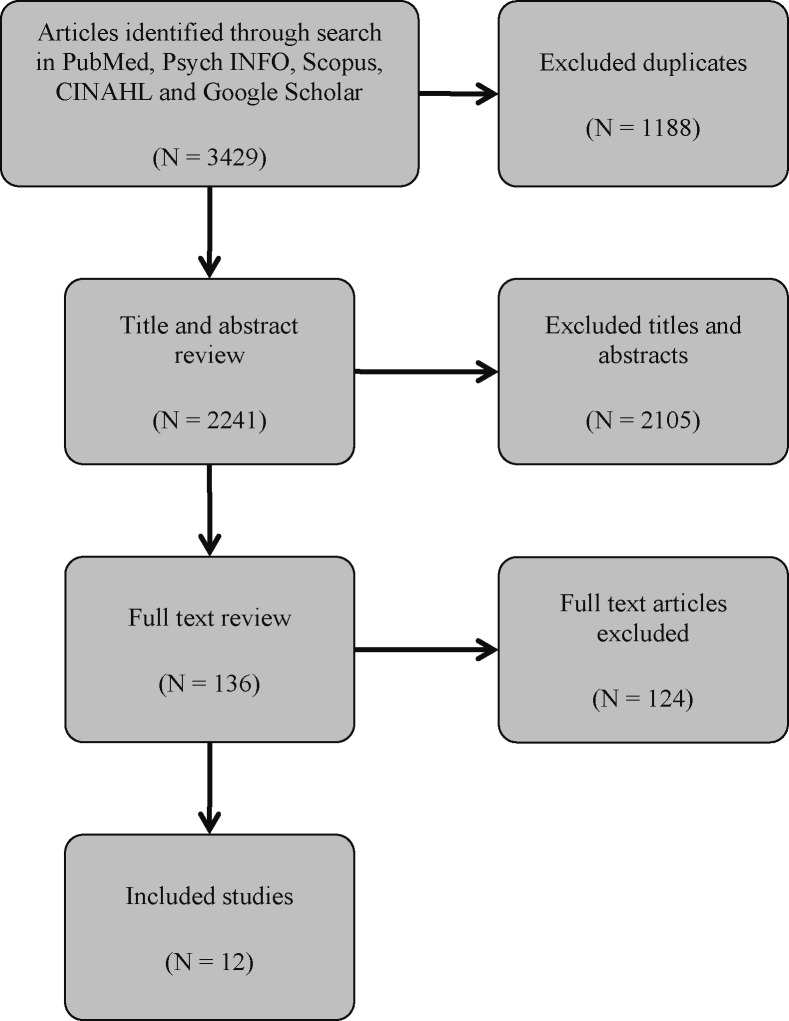
PRISMA flow-chart.

The search of databases produced 3,429 results. Following the exclusion of duplicates, another 2,241 studies were excluded during the title and abstract screening. This resulted in 136 articles, the full paper texts of which were retrieved and reviewed. The search through citations did not reveal any additional relevant sources. Twelve articles, which met the inclusion criteria, were eventually included (**Figure 1**).

### Characteristics of Included Studies

We have included 12 RCTs (published from 1992 to 2015) in our systematic review with a total number of 958 patients. Patients’ age ranged from 6 to 18 years with reported means ranging from 11 ± 3 to 15 ± 2.4 years. Two studies (18, 24) implemented Independent Medical Examination (IME), and seven studies used intention to treat (ITT) analysis. Only three studies (25–27) did not proceed with the ITT method due to high rate of trial completion (92.9%, 100%, and 97.5%, respectively). The pre-trial treatment of participants was markedly heterogeneous, wherever reported. One of the trials was of a crossover design (25), and only data extracted from initial 8 weeks were included in this review (the second phase had marked dropout rates). One study (28) directly (without a placebo arm) compared the two SSRIs (fluoxetine versus citalopram), and the extracted data were only used for evaluating the efficacy of different SSRIs.

All included studies reported primary outcome measures using CY-BOCS or calculations based on it. Ten studies reported rates of response to treatment, the definition of which varied across studies: 4, 2, and 1 studies defined response as 25%, 30%, and 40% decrease from baseline CY-BOCS, respectively, one study used end-treatment CY-BOCS ≤16 as a definition, and in only one report (29) the authors did not specify their definition albeit reported a rate. The rationale for combining such heterogenous dichotomous outcomes in a meta-analysis was that patients qualifying for a larger percent reduction (e.g., 40%) also simultaneously qualify for lesser percent reductions (e.g., 25% or 30%); thus one could be considering all “responding” patients to at least qualify for the least reported percent reduction. Three studies reported rates for CY-BOCS ≤11, which was defined as remission in all three. One study (26) did not specifically report the above-mentioned rates, but the raw data were included in the published article, which allowed appropriate calculations to be made.

Altogether 39.5% of patients involved in the 12 RCTs responded to all forms of treatment (intervention and placebo) as defined by the variety of numerical cutoffs (25%, 30%, and 40% CY-BOCS reduction, CY-BOCS ≤16 and not otherwise specified), and 6.4% were considered to have achieved remission (as defined by CY-BOCS ≤11). The characteristics of included studies and their participants are presented in further detail in **Tables 1** and **2**, respectively. **Table 3** presents the risk of bias assessments across all included studies.

**Table 1:** Characteristics of included studies.

Study*	Diagnosis based on DSM	Pre-trial treatment	Intervention	Control	SSRI	Dose (mg)	Treatment Duration (weeks)	Reported Outcome***	ITT
Alaghband-Rad and Hakimshooshtary (28)	IV	None	SSRI (a)	SSRI (b)	Fluoxetin, Citalopram	20	6	mean	+
Asbahr et al. (27)	IV	no trial’s treatment in prior 6 months	SSRI	CBT	Sertraline	≤ 200	12	mean, 25% red	-
Geller et al. (30)	IV	no crit.	SSRI	Placebo	Fluoxetine	20–60	13	mean	+
Geller et al. (31)	IV	no crit.	SSRI	Placebo	Paroxetine	10–50	10	change, 40% red	+
Liebowitz et al. (29)	III-R	no trial’s treatment	SSRI	Placebo	Fluoxetine	60–80	8	mean, 25%red	+
March and Friesen (32)	III-R	no trial’s treatment	SSRI	Placebo	Sertraline	≤200	12	mean, 25%red	+
Neziroglu et al. (26)	IV	failed CBT	SSRI+CBT	SSRI	Fluvoxamine	≤200	42	mean	-
POTS (4)	IV	no trial’s treatment	SSRI	Placebo	Sertraline		12	mean, rem	+
			CBT	Placebo		75–200			
			SSRI+CBT	Placebo					
Riddle et al. (25)**	III-R	no trial’s treatment	SSRI	Placebo	Fluoxetine	20	8	mean	-
Riddle et al. (33)	III-R	no crit.	SSRI	Placebo	Fluvoxamine	50–200	10	change, 25%red mean,	+
Skarphedinsson et al. (18)	IV	none in prior 6 months	SSRI	CBT	Sertraline	≤200	16	30%red, CYBOCS ≤ 16 mean,	+
Storch et al. (24)	IV	no trial’s treatment	regular SSRI+CBT	Placebo + CBT	Sertraline	≤200	18	30%red, rem	+
			titrated SSRI+CBT	Placebo + CBT					

*All were RCTs.

**A crossover trial.

***All related to CY-BOCS score: mean = end - treatment mean score, change = change from baseline score; red = reduction from baseline score, rem = remission (i.e. end treatment CY-BOCS ≤11). Where missing in the articles, the data were either calculated from other reported values or, where appropriate, previous meta-analyses were consulted. ITT, intention to treat.

**Table 2:** Characteristics of participants from included studies.

Study	N (size)	Arms	N (arms)	Male %	Mean age (SD)	Mean age at onset	Baseline CY-BOCS	Completed the study (%)	Responders* (%)	Remission** (%)
Alaghband-Rad and Hakimshooshtary (28)	29	Fluoxetine	15	58.6	14 ± 2.4	NR	26.7 ± NR	82.8	NR	NR
		Citalopram	14				28.0 ± NR		NR	NR
Asbahr et al. (27)	40	SSRI	20	65.0	13 ± 2.5	9 ± 3.2	27.0 ± 6.7	97.5	90.0	NR
		CBT	20				26.3 ± 4.9		95.0	NR
Geller et al. (30)	103	SSRI	71	47.6	11 ± 2.9	NR	24.5 ± 5.1	67.0	49.3	NR
		Placebo	32				26.3 ± 4.6		25.0	NR
Geller et al. (31)	203	SSRI	98	57.6	11 ± 3	8 ± 3.1	24.4 ± 5.0	71.4	62.2	NR
		Placebo	105				25.3 ± 5.1		40.0	NR
Liebowitz et al. (29)	43	SSRI	21	58.1	13 ± 2.7	NR	22.5 ± 4.2	88.4	57.1	NR
		Placebo	22				23.8 ± 5.8		31.8	NR
March and Friesen (32)	187	SSRI	92	NR	13 ± NR	8 ± NR	23.4 ± 4.6	83.4	53.3	NR
		Placebo	95				22.2 ± 6.2		36.8	NR
Neziroglu et al. (26)	10	SSRI + CBT	5	60.0	15 ± 2.4	10 ± NR	28.0 ± 5.6	100.0	40.0 / 80.0*	0.0
		SSRI	5				22.8 ± 3.8		0.0 / 20.0*	0.0
POTS (4)	112	CBT	28	50.0	12 ± 2.7	NR	26.0 ± 4.7	86.6	NR	39.3
		SSRI	28				23.5 ± 4.7		NR	21.4
		SSRI + CBT	28				23.8 ± 3.0		NR	53.6
		Placebo	28				25.2 ± 3.3		NR	3.6
Riddle et al. (25)**	14	SSRI	7	42.9	12 ± 2.3	NR	24.3 ± 4.2	92.9	NR	NR
		Placebo	7				20.2 ± 7.7		NR	NR
Riddle et al. (33)	120	SSRI	57	53.3	13 ± NR	9.4 ± NR	24.2 ± 4.4	61.7	42.1	NR
		Placebo	63				24.2 ± 4.8		27.0	NR
Skarphedinsson et al. (18)	50	SSRI	22	48.0	14 ± 2.7	NR	21.1 ± 3.7	72.0	45.5 / 45.5*	27.3
		CBT	28				21.3 ± 4.0		35.7 / 50.0*	32.1
Storch et al. (24)	47	reg SSRI + CBT	14	61.7	14 ± 2.7	NR	23.6 ± 4.5	70.2	57.1	42.9
		slow SSRI + CBT	17				26.7 ± 5.7		64.7	26.5
		Placebo + CBT	16				25.1 ± 4.0		62.5	18.8
TOTAL	958			43.7				76.6	39.5 %***	6.4

NR, not reported.

Where missing in the articles, the data were either calculated from other reported values or, where appropriate, previous meta-analyses were consulted.

*Responders were defined differently across studies: 25% reduction of CY-BOCS from baseline (27, 31–33), 30% reduction from baseline (18, 24) 40% reduction from baseline (30), end-treatment CY-BOCS ≤ 16 (18), and not otherwise specified (29). Neziroglu et al. (26) did not specifically report response and remission rates, which were calculated from the raw data and 25 % or 30% reductions/CY-BOCS ≤16 are presented in the corresponding row. 30% reduction/CY-BOCS ≤16 are presented for the row of Skarphedinsson et al. (18).

**Remission was defined as CY-BOCS ≤11.

***Only 30% reduction rates were taken into account for Neziroglu et al. and Skarphedinsson et al.

**Table 3:** Risk of bias assessment.

Study	Random Sequence Generation	Allocation Concealment	Blinding of Participants/Personnel	Blinding Outcome Assessors	Incomplete Outcome Data	Selective Reporting	Other Bias
Alaghband-Rad and Hakimshooshtary (28)	?	?	?	?	-	-	-
Asbahr et al. (27)	?	?	-	-	+	+	+
Geller et al. (30)	?	?	?	?	+	+	+
Geller et al. (31)	+	?	?	?	-	+	-
Liebowitz et al. (29)	?	?	?	+	+	+	+
March and Friesen (32)	+	?	+	?	+	-	+
Neziroglu et al. (26)	?	?	-	?	+	+	+
POTS (4)	+	+	?	+	?	+	+
Riddle et al. (25)**	?	+	+	?	+	+	+
Riddle et al. (33)	?	?	+	+	+	+	+
Skarphedinsson et al. (18)	+	+	-	?	+	+	+
Storch et al. (24)	+	+	?	+	+	+	+

“+” is of low risk; “-”is of high risk; “?” is of unclear risk.

Since the meta-analyses performed in this review included less than 10 studies (see SSRI vs. placebo, N = 7) and were constructed on random effects model, funnel plots, and trim and fill analyses [see Refs. (34, 35)] would not be useful to assess publication bias. Instead, the file drawer phenomenon was recognized, and the possibility of publication bias was tested *via* Orwin’s fail-safe N formula [see below and Ref. (36)].

$$N_{fs}=\frac{N_{0}({\bar{d}}_{0}-{\bar{d}}_{c})}{d_{c}-d_{fs}}$$

Because the only meta-analysis performed in this review that utilizes change from baseline for comparison was that for SSRI vs. placebo, the decision was made to calculate N_fs_ only with data from that analysis. The other analyses in this review used end-treatment scores for comparison, which by far is not the most appropriate manner of demonstrating effect size and is not recommended by Cochrane Handbook for Systematic Reviews of Interventions (version 5.1.0).

However, the following values were inserted into the formula above: N_0_ = 7, d_0_ = −0.43, d_c_ = 0.2 (since the effect size of the analysis, by the rule of thumb, was designated as small), and d_fs_ = 0.

The calculation followed that N_fs_ = 8 studies with reported CY-BOCS standardized mean difference of 0.2 (small effect size, opposite direction) were required to bring the current SMD from −0.43 to 0.

## Comparisons

### Monotherapy With SSRI Versus Placebo

**Figure 2:**
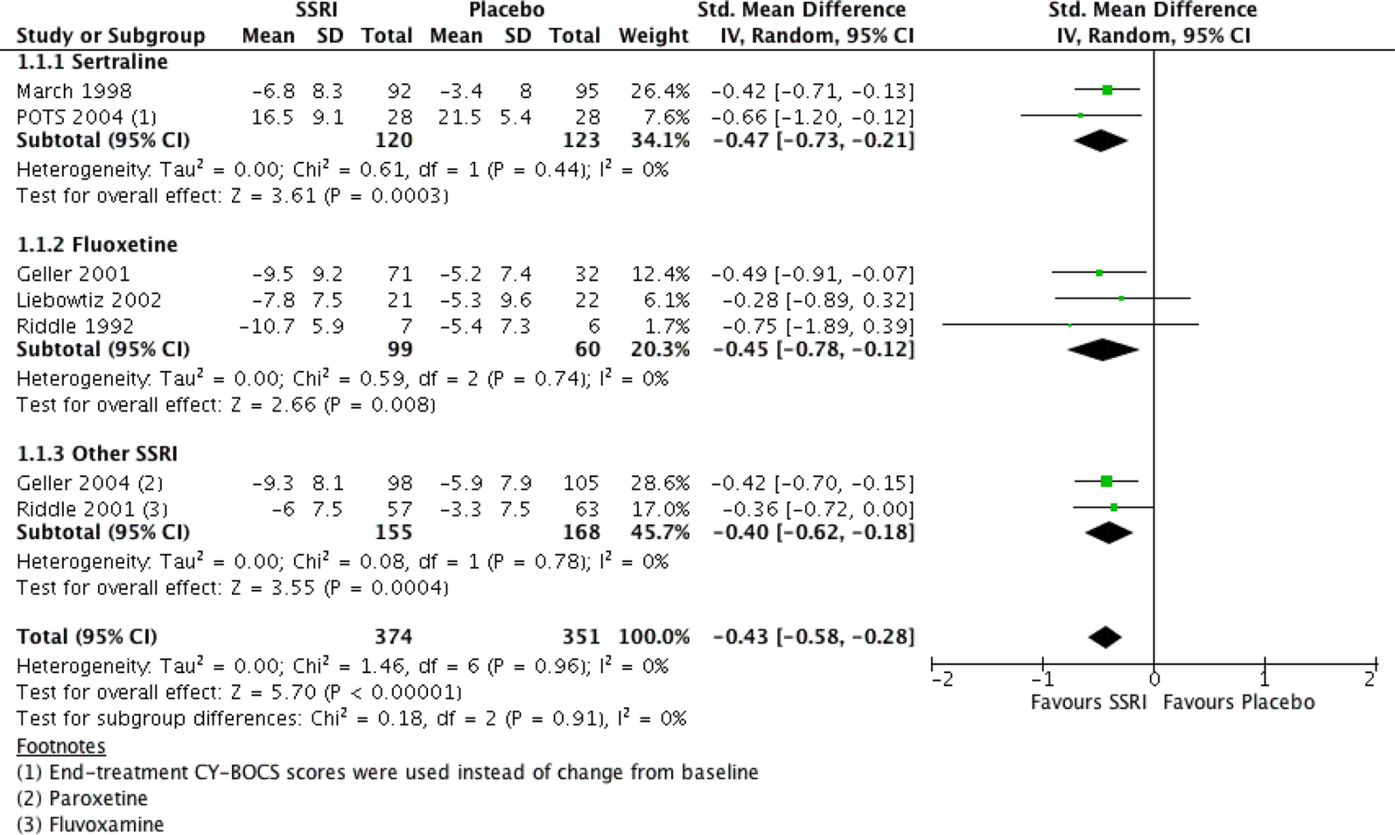
SSRI vs. placebo. Standardized mean differences of change from baseline CY-BOCS.

There were seven RCTs comparing SSRIs with placebo treatment included in our review. One of them (25) was conducted in a crossover fashion and only data from the first phase (first 8 weeks) of the study were extracted and analyzed. The reason for this decision was that after the crossover, 50% of initially enrolled patients dropped out for reasons such as recurrence of symptoms after switching to placebo, and the sample size became too small. For similar reasons and also to yield homogenous treatment durations only data from the “acute phase” (i.e., the first 8 weeks) of another study (29) were incorporated into our analysis (the study also presented data from a later “maintenance phase,” which lasted for another 8 weeks and was performed with only participants who were responders). Another study (4) compared four intervention arms (SSRI, CBT, SSRI + CBT, placebo), from which we included the data for SSRI and placebo into our analysis. Overall, we analyzed data on a total number of 725 patients, 51.6% and 48.4% of which received SSRI and placebo, respectively. All studies except one (4) either reported data on mean and SD of change from baseline CY-BOCS or presented other valuable data (*t*, p, baseline, end-treatment scores), and the change and SD were calculated in the following manner: the available values were entered into Rev Man Software’s built-in calculator, which returned the desirable measures. The mean differences and their pooled analysis are shown in **Table 4**. The studies demonstrated effect sizes (SMD) ranging from small (−0.28, 95% CI, −0.9 to 0.3) to moderate (−0.75; 95% CI, −1.9 to 0.4), and the pooled analysis achieved a *small effect size* of −0.43 (95% CI, −0.6 to −0.3), which was *significant* (see **Figure 2**), favoring SSRI. The subgroup analysis did not demonstrate any difference between different SSRIs. The subgroups of sertraline, fluoxetine, and other SSRIs (paroxetine and fluvoxamine) all achieved similar small pooled effect sizes of −0.45 (95% CI, −0.73 to −0.21), −0.45 (95% CI, −0.78 to −0.12), and −0.40 (95% CI, −0.62 to −0.18), respectively, which did not differ significantly (Chi^2^ = 0.18, df = 2 (P = 0.91), I^2^ = 0%).

**Table 4:** SSRI vs Placebo.

Name	Size	SSRI	Mean difference [95% CI]
Geller et al. (30)	103	Fluoxetine	−4.30 [−7.64 to −0.96]
Liebowitz et al. (29)	43	Fluoxetine	−2.50 [-7.64, 2.64]
Riddle et al. (25)	14	Fluoxetine	−5.30 [-12.60, 2.00]
March and Friesen (32)	187	Sertraline	−3.40 [-5.74, -1.06]
POTS (4) *	56	Sertraline	−5.0 [−8.92 to −1.08]*
Geller et al. (31)	203	Paroxetine	−3.40 [−5.60 to −1.20]
Riddle et al. (33)	120	Fluvoxamine	−2.70 [−5.39 to −0.01]
Total	669**		−3.38 [−4.60 to −2.16]**

Mean differences of change from baseline CY-BOCS.

*The change from baseline SD could not be retrieved or otherwise calculated for POTS (4); thus, mean difference of end-treatment CY-BOCS was provided.

**The pooled size and mean difference were calculated omitting POTS (4) data.

Five studies also reported response rates, and one study reported remission rates based on various definitions (see above and **Table 5**). The pooled ln OR for response rate was 0.8 (95% CI, 0.5–1.1), which converts to SMD of 0.5, 95% CI 0.2, 0.8, p < 0.05 (37) yielding a significant moderate effect size and favoring SSRI (37).

**Table 5:** SSRI vs Placebo. Response and remission rates.

Study	Definition	Size (N)	ln OR, [95% CI]	P value
Response rates				
Geller et al. (30)	40% reduction	103	1.1 [0.2, 2.0]	0.02
Liebowitz et al. (29)	NOS	43	0.9 [0.3, 1.5]	0.002
March and Friesen (32)	25% reduction	187	1.1 [−0.2, 2.3]	0.1
Geller et al. (31)	25% reduction	203	0.7 [0.1–1.3]	0.02
Riddle et al. (33)	25% reduction	120	0.7 [−0.1 to 1.4]	0.08
Total		656	0.8 [0.5–1.1]	<0.05
Remission rates				
POTS	CY-BOCS ≤11	56	2.0 [−0.2 to 4.2]	0.07

### Monotherapy With SSRI Versus CBT

**Figure 3:**
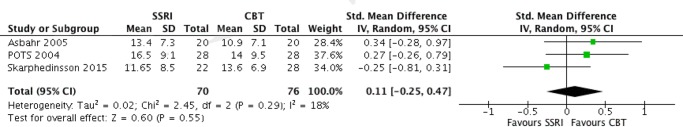
SSRI vs CBT. Standardized mean differences based on end-treatment CY-BOCS.

We have identified three RCTs comparing these two treatment modalities, all utilizing sertraline. In one study where the author’s randomized patients to either SSRI or CBT (18), all patients were pre-treated with CBT for 13 weeks before randomization, and thus switching to SSRI was compared to continued CBT over 16 weeks duration. Another study compared group CBT (GCBT) and SSRI treatment during 12 weeks (27). The third study (4), as mentioned above, separately compared three intervention arms with placebo. Data extracted from the SSRI and CBT arms were used for comparison. The SDs of change from baseline were not available for all three studies; thus, comparison was made utilizing the end treatment CY-BOCS scores. The two studies (4, 27) showed a mean difference of 2.5 (95% CI, –1.96 to 6.96) and 2.5 (95% CI, –2.37 to 7.37) in favor of CBT with small effect sizes: SMD = 0.34 (95% CI, –0.28 to 0.97) and 0.27 (95% CI, –0.26 to 0.79), respectively, which *were not significant*. The study by Skarphedinsson et al. favored SSRI with a mean difference of –1.95 (95% CI, –6.33 to 2.43) and a small effect size of –0.25 (95% CI, –0.81, 0.31), which was not significant (18). The pooled analysis of the three studies found a mean difference of 0.92, 95% CI: −2.05, 3.88, g = 0.11, 95% CI: −0.25, 0.47, which was not significant albeit weakly favoring CBT (see **Figure 3**). The studies were also compared with regard to their response and remission rates (see **Table 6**). Asbahr et al. and Skarphedinsson et al. reported response rates as defined by 25% and 30% reductions from baseline CY-BOCS scores, respectively (18, 27). The ln OR for the two studies were –0.7 (95% CI, –3.2 to 1.7) and 0.4 (95% CI –0.7 to 1.5), respectively. The combined ln OR was 0.2 (95% CI, –0.8 to 1.2), weakly favoring SSRI. Skarphedinsson et al. and POTS presented remission rates of ln OR = –0.2 (95% CI, –1.5 to 1.0; and –0.9 (95% CI, −2.0 to 0.3); respectively, as defined by end-treatment CY-BOCS ≤11 (4, 18). The combined ln OR was −0.6 (95% CI, −1.4 to 0.3), which weakly favors CBT. Both comparisons were statistically non-significant (P > 0.05).

**Table 6:** SSRI vs CBT. Response and Remission Rates.

**Study**	**Definition**	**Size (N)**	**ln OR, [95% CI]**	**P value**
*Response rate*				
Asbahr et al. (27)	25% reduction	40	−0.7 [−3.2 to 1.7]	0.6
Skarphedinsson et al. (18)	30% reduction	50	0.4 [−0.7 to 1.5]	0.5
Total		90	0.2 [−0.8 to 1.2]	0.5
*Remission rate*				
Skarphedinsson et al. (18)	CY-BOCS ≤11	50	−0.2 [−1.5 to 1.0	0.7
POTS (4)	CY-BOCS ≤11	56	−0.9 [−2.0 to 0.3]	0.15
Total			−0.6 [−1.4, 0.3]	0.2

### Combined Therapy With SSRI and CBT Versus Placebo

From studies included in our review, only one study (4) undertook a comparison of combination treatment versus placebo. The SD values of change from baseline could not be extracted from the paper or retrieved otherwise. Thus, only the end-treatment mean CY-BOCS could be compared.

The combination treatment and placebo arms yielded scores of 11.2 (SD = 8.6) and 21.5 (SD = 5.4), respectively, and the mean difference (MD) of these scores *demonstrated the combination to be significantly superior* (MD = −10.3, 95% CI, −14.1 to −6.5) compared with placebo (SMD = −1.41, 95% CI, −2.0 to −0.82). The paper also provided data regarding remission rates as defined by achieved CY-BOCS ≤11 with odds ratio of 31.1 (ln OR = 3.4; 95% CI, 1.3–5.6) and 50% risk difference, which were *statistically significant* (P < 0.05).

### Combined Therapy With SSRI and CBT Versus CBT Alone

Only two studies (4, 24) presented outcomes for comparing SSRI and CBT combination and CBT monotherapy. Both studies used sertraline as the intervention medication and reported outcomes as end-treatment CY-BOCS scores. In the study by Storch et al., CBT was combined with placebo, and not used as actual monotherapy (24). The study involved two arms: one with regular dosing and the other with titrated dosing. The titration arm was used to compare to the POTS study, which only used the dose titration approach.

In the study by Storch et al., a mean difference of −0.2 (95% C,: −6.2 to 5.8) was observed between the regular and CBT + placebo arms with an effect size of −0.02, 95% CI: −0.74, 0.69 (24). The titration arm showed MD = 1.6; (95% CI, −3.3 to 6.5; SMD, −0.2; 95% CI, −0.5 to 0.9). Both findings were non-significant. The POTS study achieved a mean difference of −2.8 (95% CI, −7.6 to 2.0), which was of small effect size, albeit insignificant (SMD = −0.3, 95% CI, −0.83 to 0.22) (4). A pooled analysis of the data from POTS and titration arm of Storch et al., utilizing the random effects model (as the Storch et al. study did not actually use CBT monotherapy but CBT + placebo) achieved a mean difference of −0.6 (95% CI, −4.9, 3.7) and effect size of −0.1 (95% CI, −0.6, 0.4, thus *demonstrating no significant difference* (4, 24).

### Combined Therapy With SSRI and CBT Versus SSRI Alone

Two studies (4, 26) compared the combination of SSRI and CBT with SSRI monotherapy. Both reported end-treatment CY-BOCS data. The study by Neziroglu et al. specifically compared the combination treatment with monotherapy in a population that has previously failed CBT monotherapy (26). The authors evaluated the benefit of adding CBT on SSRI treatment regimen. Raw data were available and included the end-treatment CY-BOCS for baseline, 10, 43, and 52 weeks, and at 2 years follow-up, and change scores. To encompass homogenous treatment durations the comparison was made using data from the 52nd week. A mean difference of −5.4 (95% CI, −11.3, 0.5 was observed with a significant, large effect size of SMD, −1.0, (95% CI: −2.4 to 0.4). The POTS study yielded a mean difference of −5.3, 95% CI: −10.0, −0.7 with a moderate effect size of −0.6, 95% CI: −1.1, −0.1, which was significant (4). The pooled analysis of data from both studies comprising a total of 66 patients achieved a mean difference of −5.3 (95% CI, −9.0 to −1.7), which was significant and of moderate effect size (SMD = −0.7, 95% CI: −1.1, −0.2), thus *favoring the combination treatment.*

### Comparison of SSRI Versus No SSRI Intervention Arms

**Figure 4:**
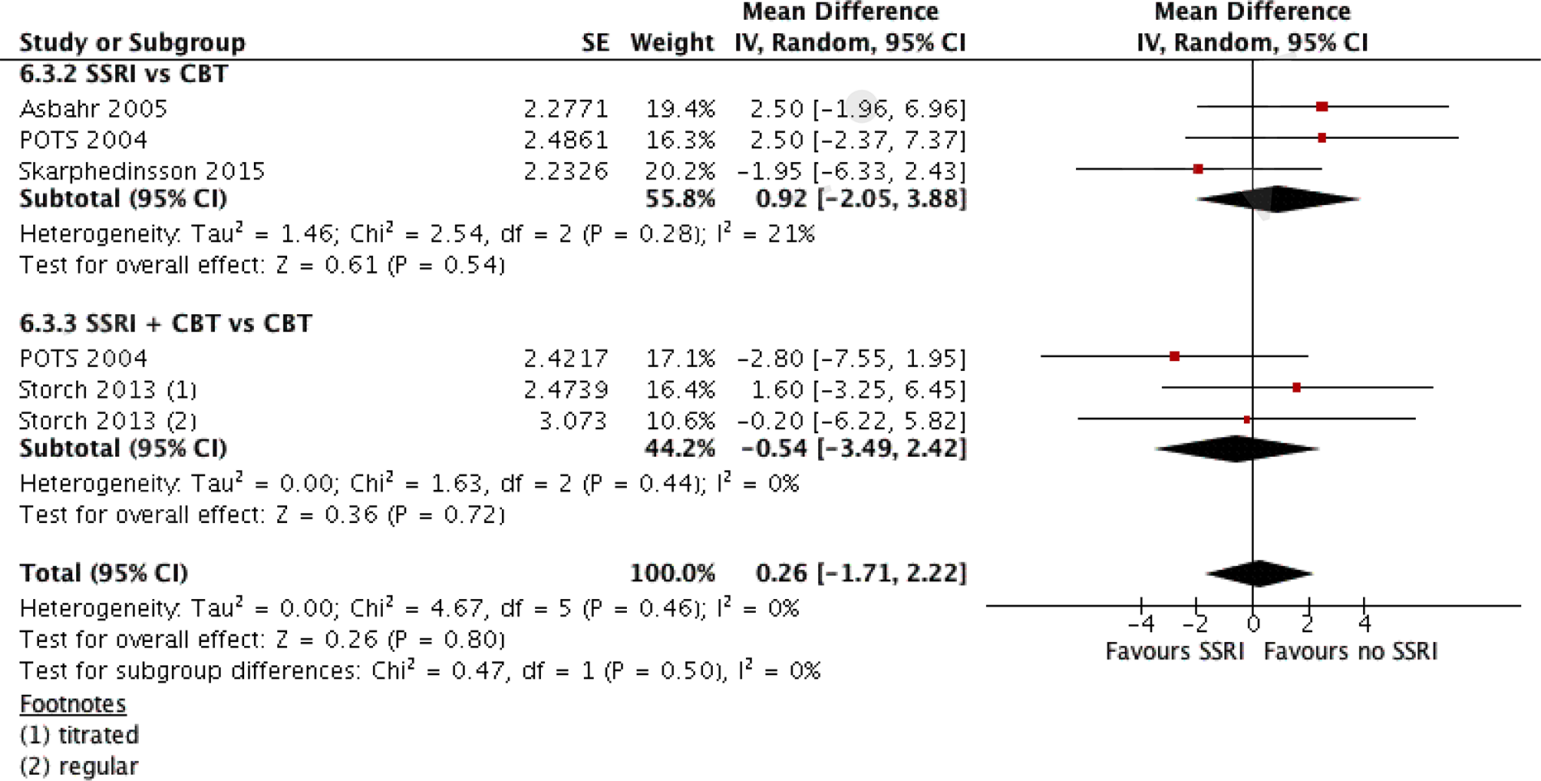
Comparison of interventions with SSRI and with no SSRI. A generic inverse variance analysis.

Considering placebo to be not an “actual intervention,” it was suggested that a comparison could be made between treatment arms that did or did not use SSRI. Four studies suitable for such analysis were reported [Refs. (4, 18, 24, 27)]. All four used sertraline as the SSRI of intervention. Three compared SSRI and CBT monotherapies, and two compared the combination of SSRI and CBT with CBT monotherapy (see **Table 7**). A pooled analysis of the mean differences using generic inverse variance and random effects analysis model demonstrated *no significant difference between the two interventions* (MD = 0.26, 95% CI, −1.7 to 2.2) (see **Figure 4**).

**Table 7:** Effect sizes for studies comparing SSRI versus no SSRI interventions.

Name	Size	SSRI	Effect size: SMD [95% CI]
SSRI vs CBT			
Asbahr et al. (27)	40	Sertraline	0.34 [-0.28, 0.97]
POTS (4)	56	Sertraline	0.27 [-0.26, 0.79]
Skarphedinsson et al. (18)	50	Sertraline	-0.25 [-0.81, 0.31]
SSRI + CBT vs CBT			
POTS (4)	56	Sertraline	-0.30 [-0.83, 0.22]
Storch et al. (24)	33*	Sertraline, titrated	0.22 [-0.47, 0.90]
Storch et al. (24)	30*	Sertraline, regular	-0.02 [-0.74, 0.69]
SSRI vs Placebo			
Geller et al. (30)	103	Fluoxetine	-0.49 [-0.91, -0.07]
Geller et al. (31)	203	Paroxetine	-0.42 [-0.70, -0.15]
Liebowitz et al. (29)	43	Fluoxetine	-0.28 [-0.89, 0.32]
March and Friesen (32)	187	Sertraline	-0.42 [-0.71, -0.13]
POTS (4)	56	Sertraline	-0.66 [-1.20, -0.12]
Riddle et al. (25)	14	Fluoxetine	-0.75 [-1.89, 0.39]
Riddle et al. (33)	120	Fluvoxamine	-0.36 [-0.72, 0.00]
SSRI + CBT vs Placebo			
POTS (4)	56	Sertraline	-1.41 [-2.00, -0.82]

*These represent summation of arms (SSRI + placebo) and both share the same placebo control group (i.e. 14 + 16 and 17 + 16).

### Comparison of SSRI Versus All No SSRI Interventions, Including Placebo

**Figure 5:**
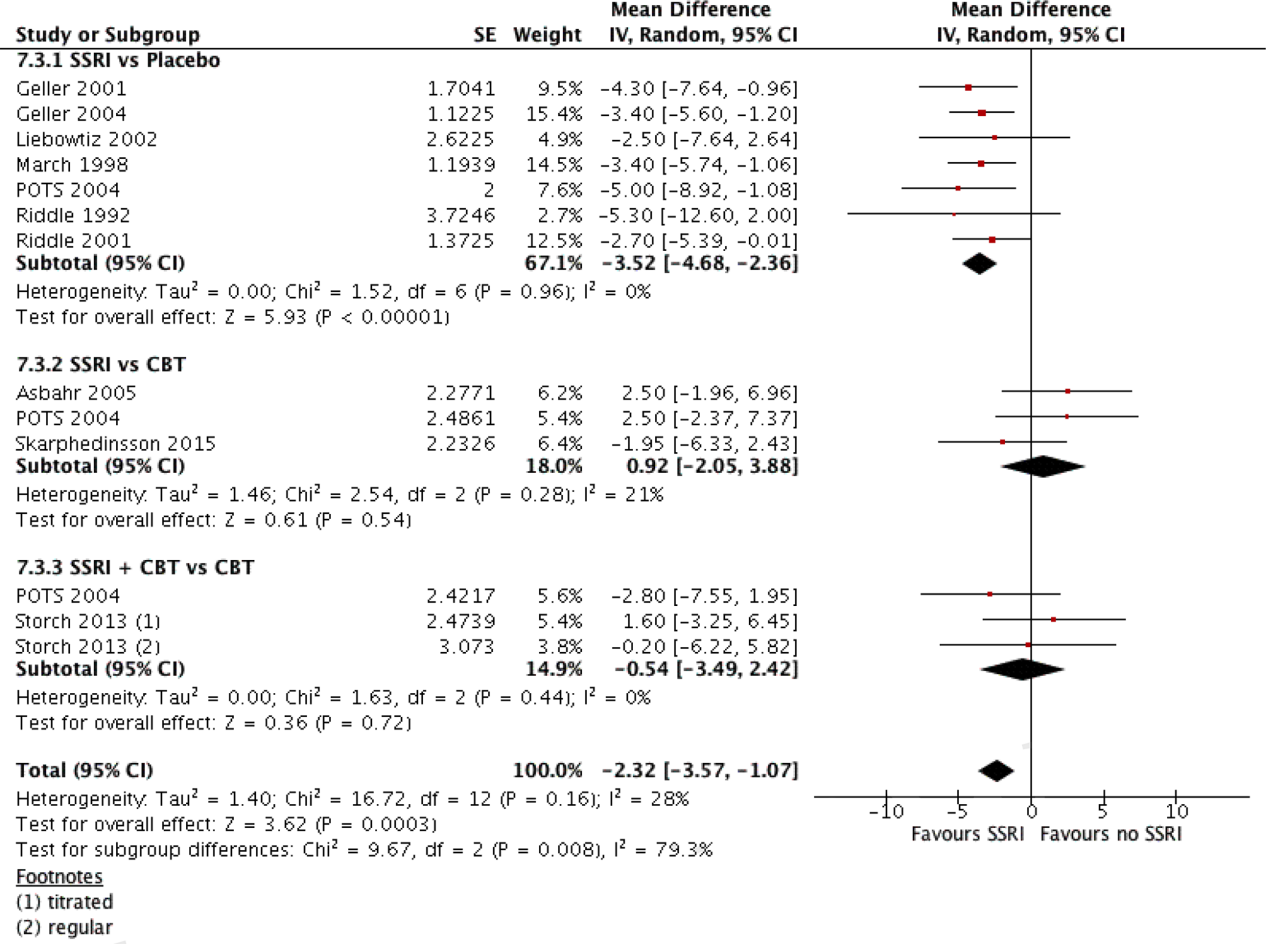
Comparison of interventions with SSRI versus all interventions without SSRI, A generic inverse variance analysis.

Contrary to the comparison above, another suggestion was made to make a comparison in a similar fashion but including placebo as an “intervention” (see later in the discussions). Data from SSRI vs. Placebo comparison were added to the previous generic inverse variance analysis. The SSRI + CBT vs. Placebo arm of POTS study was not included in the analysis, as having a large effect size (SMD = −1.4, 95% CI: −2.0, −0.8) would clearly deviate the results. The pooled analysis demonstrated a mean difference of −2.3, 95% CI, −3.6 to −1.1, *which was of small size, albeit significant* (see **Figure 5**).

### Comparison of Different SSRIs

**Figure 6:**
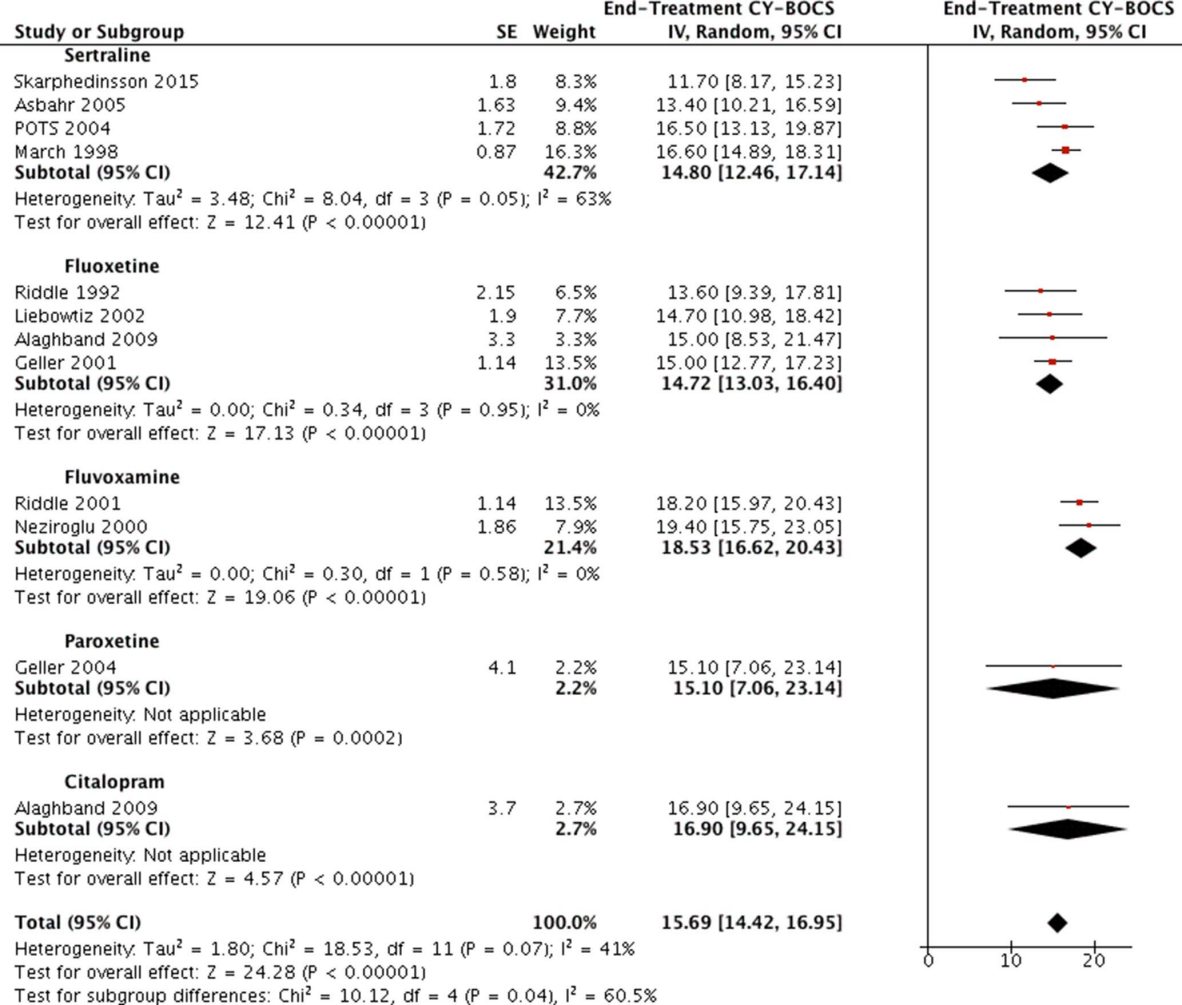
Comparison of SSRI end-treatment CY-BOCS scores. A generic inverse variance analysis.

None of the identified studies utilized head-to-head design to directly compare outcomes of different SSRI treatments. Data of end-treatment CY-BOCS scores from all available studies reporting a treatment arm of SSRI monotherapy were compared using generic inverse variance and random analysis model (see **Figure 6**). Four studies were using sertraline (4, 18, 27, 32), four studies using fluoxetine (25, 28–30), two studies using fluvoxamine (26, 33), one study using paroxetine (31), and one study using citalopram (28). The standard deviations or errors were reported in all articles except in Alaghband et al., for which the reported p values (<0.001 for both arms) were used to ascertain the possible *t* values from the *t* tables and to calculate the standard errors with Rev Man calculator (28). The estimated *t* and p values for fluoxetine and citalopram were *t* = 4.54, p = 0.0005 and *t* = 4.57, p = 0.0005, respectively. These converted to standard errors of SE = 3.3 and SE = 3.7, respectively.

The pooled results of studies that used the same SSRI were compared visually, and any gross differences (exceeding 2 points on CY-BOCS) were evaluated *via* the I2 statistic (which is incorporated in Rev Man). This was facilitated by unchecking all the other subgroups not to be compared. The two largest subgroups, namely, the sertraline and fluoxetine, encompassing four studies each, appeared to achieve similar results (end-treatment mean CY-BOCS = 14.8 (95% CI, 12.5–17.1; and 14.7, 95% CI: 13.0, 16.4; respectively). The largest grossly visualized difference was detected between subgroups of fluoxetine and fluvoxamine (14.7, 95% CI: 13.0, 16.4; 18.5; 95% CI, 16.6 to 20.4; respectively), mandating an evaluation of heterogeneity between them. Once the other subgroups were unchecked from Rev Man, the test for subgroup differences displayed as: Chi^2^ = 8.64, df = 1 (P = 0.003), I^2^ = 88.4%, where the I^2^ statistic notifies of *considerable heterogeneity*, suggesting a possibility of fluoxetine’s superiority. Similar findings were noted when attempting to compare sertraline and fluvoxamine in the same manner, again suggesting Sertraline’s superiority [Chi^2^ = 5.87, df = 1 (P = 0.02), I^2^ = 83.0%]. The I^2^ statistic displayed 0 for all other possible subgroup comparisons carried out in the same fashion.

## Discussions

### Summary of Main Results SSRI Versus Placebo

SSRIs are clearly effective compared to placebo for treating OCD in adolescents and children, but the effect size is small (SMD = −0.43), which converts to 3.5-point reduction in the CY-BOCS score. The clinical implication of this finding is hard to evaluate, as even though the change is statistically significant, some 3.5 points reduced from the initial CY-BOCS of 25 is merely little symptom reduction to a score of 21.5, which by no means can be designated as response (in this scenario such reduction constitutes 14% and grossly differs from those defined in the literature, e.g. 25%, 30%, 40% reduction or CY-BOCS ≤16). Since the RCTs were conducted in a mean time period of 8 weeks, the observed effect represents only the short-term finding. *Further studies with longer trial durations are required to assess the long-term effects and benefits of SSRI monotherapy.*

Using the method put forward by Chinn (37), the pooled response rate transforms to SMD of 0.5, which in contrary to that seen in continuous outcomes, is already of moderate effect size. Summarizing the findings of dichotomous outcome comparison, it suffices to note, that the implicated definitions of 25%, 30%, or 40% reductions do not necessarily mean clinically significant symptom reduction but represent more conservative cutoffs to define minimal observable change (37). By far, the achieved 20% absolute risk reduction (RD) converts to a number needed to treat (NOT) of five patients, which could be interpreted as follows: one would need to treat five patients to achieve minimal clinically noticeable change in a single patient. Similar to response rates are those regarding remission, as defined CY-BOCS ≤11. Although reported in only one study, the absolute risk difference of remission was 18%, which converts to NNT = 6 patients, which is somewhat consistent with that of response, adding a valuable notice, which for each six treated patients, one will respond and another one will probably experience remission. The lack of studies evaluating remission rates in SSRI vs. Placebo design limits the value of the later statement. The observed effect size for children and adolescents is somewhat similar to those reported in treatment trials of adults with OCD (38).

The reported findings across studies are overall reliable, as judged from risk of bias, albeit heterogeneous. The interpretations regarding the effectiveness of SSRI versus Placebo presented above are mainly consistent with those previously stated (21, 22, 39).

Future studies with the mentioned design are less probable, the reason being twofold. First, being superior over placebo does not yet advocate for the intervention’s clinical implications, especially when an even more effective treatment option clearly exists. Such an option, exemplified herein by CBT, has been fairly validated, and its efficacy in children and adolescents has been confirmed multiple times by various studies (11, 20, 21, 40–42). Second, leaving the “placebo” arms of children untreated for the study duration would be somewhat unethical. It is generally considered, that a comparison of this or that intervention with the current “gold standard” treatment would be far more appropriate in such settings. This has been clearly stated in the clinical epidemiology textbook by Haynes (43). It is expected that further research examining the efficacy of SSRIs in OCD treatment would more commonly use SSRI vs. CBT design, or evaluate the added benefit of SSRIs (e.g., SSRI + CBT vs. CBT), as discussed below.

### The Effectiveness of SSRI in Other Comparisons

The only study comparing the combination of SSRI and CBT with placebo (4) found the former to be highly effective, with a mean CY-BOCS reduction of 10, which in clinical practice obviously would make a difference: bringing the CY-BOCS of a patient from 25 to 15 would denote both clinically important symptom reduction and match the definition of “response,” albeit not that of “remission” (4). Also, a 50% risk reduction was observed for remission, which transforms into NNT = 2. Despite these findings, only the end-treatment scores were compared instead of change scores. Thus, the validity of the finding cannot be ascertained. Besides, clinicians do not usually prescribe placebos to proudly state that the combination is superior to it, neither is the comparison of any value, as both CBT and SSRI are interventions and both could have contributed to the change, and the study cannot distinguish the input effect of each. As stated above, few studies of such designs are expected in the future.

There were two studies comparing the combination to SSRI monotherapy (4, 26) and two studies comparing it to CBT monotherapy (4, 24). A significant benefit was observed when adding CBT to SSRI treatment (with a mean five-point difference in CY-BOCS), but no significant difference after adding SSRI to CBT treatment. Both findings point to the superiority of CBT monotherapy and question the value of adding SSRI to the already effective treatment regimen with no additional benefit. In contrast to these findings, the pooled analysis of the three trials with a head-to-head comparison of SSRI and CBT (4, 18, 27) did not find any significant difference between the treatment options, albeit two studies weakly favored CBT, and one weakly favored SSRI. All three demonstrated small effect sizes. These findings raise the possibility that SSRI and CBT could be equally effective options, to begin with, but if the initial treatment is chosen to be CBT, adding an SSRI regimen would not have additive effect, although if the SSRI was the option to begin with, adding CBT sessions would be beneficial to the already “responsive” patient.

The limitation regarding trial duration is as well relevant here, and the question “which treatment option would hold the benefit for the longest period” is of important clinical implication.

The findings summarized above are mainly consistent with observations from adult studies (23). We conclude that SSRI and CBT could be equal options regarding initial effectiveness, which somewhat differs from the previous meta-analysis (39), although we appreciate the large effect sizes that CBT contributes to outcomes in various study designs.

### Overall Effectiveness of SSRIs

To evaluate the overall effect of SSRIs across various treatment regimens, the effect sizes of all comparisons made between arms that involved an SSRI and those without an SSRI were entered into a generic inverse variance analysis. At first, placebos were not considered, and the overall benefit of SSRIs across four studies [6 comparisons, 2 intervention arms in each of Storch et al. (24); and POTS (4)] did not reach significance, but after adding the studies comparing SSRI to placebo, a small, albeit significant difference was noted (4, 24). The interpretation of such findings is difficult. The authors concluded that the overall effectiveness is small and is mostly seen in studies comparing to placebo. The findings are consistent with the common clinical scenario, where prescribing an SSRI to a child with OCD, who is already receiving CBT, does not improve symptoms significantly over the course of 8 weeks and continued CBT.

### Differences in SSRI Effectiveness

It suffices to say that the method implemented here was bizarre and nowhere near a head-to-head comparison or a network meta-analysis, but it was worth entertaining the idea that one SSRI could be superior over another (e.g., fluoxetine vs. fluvoxamine), thus setting soil for future investigations and direct comparisons. Overall the findings suggested the similar efficacy of fluoxetine and sertraline and favored these two over fluvoxamine, based on considerable effect size heterogeneity. As already mentioned, this does not replace direct comparisons, which are expected to take place in the future in part by RCTs probably receiving funding by pharmaceutical companies.

## Limitations

The most important limitation is the paucity of studies incorporated into meta-analyses, which clearly prevents the use of more sophisticated research tools and publication bias reporting with funnel plots and trims and fill technique. The limitation of excluding non-English studies is also recognized.

## Conclusions

The authors conclude that the results of current systematic review and meta-analysis support the existing NICE guidelines for choosing CBT as the first line of treatment or substituting it with an SSRI for patient preference matters, as both options seem to be equally effective and the choice rather depends on patient compliance with either option. Adding CBT to current SSRI treatment is an effective option for non-responders and partial responders but adding SSRI to ongoing CBT does not hold a significant benefit. The SSRIs have different effectiveness, and their relative efficacy remains to be investigated.

## Future Research

Head-to-head studies are required to evaluate the relative efficacy of different SSRIs, and a number of new direct studies are needed to compare SSRIs and CBT. Considering the interesting outcomes of group CBT, further studies should evaluate this intervention. The reported outcomes are expected to include pre- and post-treatment CY-BOCS scores, change from baseline and respective standard deviations, response and remission rates, with more homogenous definitions. A research guide from authorities for investigating treatment in children and adolescents with OCD would be appropriate and of great help to settle such reporting discrepancies. Regulations regarding study duration would also contribute to the future research to evaluate the long-term effects of different treatment regimens, which by all means remains uninvestigated in children and adolescents with OCD.

## Author Contributions

VK, AK, GB, and RA participated in the study conception. RB, MA, AZ, and SD participated in the study design. VK, AK, RB, RA, and MA participated in the data acquisition. RA, RB, MA, and GB participated in the data analysis and interpretation. VK, AK, SD, and AZ participated in writing the paper draft and critical revision of the manuscript.

## Conflict of Interest Statement

The authors declare that the research was conducted in the absence of any commercial or financial relationships that could be construed as a potential conflict of interest
